# Design of Experiment-Assisted Development of Biodegradable Chitosan/Whey Protein Isolate Films as Advanced Pro-Regenerative Wound Dressings

**DOI:** 10.1021/acs.biomac.6c00270

**Published:** 2026-07-21

**Authors:** Caterina Valentino, Barbara Vigani, Marco Ruggeri, Angelica Pellegrini, Giampiero Pietrocola, Cinzia Boselli, Antonia Icaro Cornaglia, Giuseppina Sandri, Silvia Rossi

**Affiliations:** † Department of Drug Sciences, 19001 University of Pavia, Viale Taramelli 12, 27100 Pavia, Italy; ‡ Department of Molecular Medicine, 19001 University of Pavia, P.le Golgi 2, 27100 Pavia, Italy; § Department of Public Health, Experimental and Forensic Medicine, 19001 University of Pavia, via Forlanini 2, 27100 Pavia, Italy

## Abstract

Chitosan (CS) and whey protein isolate (WPI) are promising biomacromolecules for wound healing; however, a rational understanding of how polymer–protein interactions influence the performance of wound dressings is still lacking. Herein, CS/WPI films were developed and optimized through a Design of Experiment (DoE) approach to investigate the influence of CS molecular weight (MW) and CS:WPI ratio on polymer–protein interactions, quantified by rheological synergism. The optimized formulation (CS:WPI 1:3 with medium-MW CS) demonstrated superior tensile strength (∼2.7 MPa), higher storage modulus (∼1 × 10^5^ MPa in the dry state), and enhanced resistance to degradation (residual mass ∼50% after 7 days). These films showed the most pronounced antimicrobial activity against *S. aureus*, promoted fibroblast migration and proliferation without cytotoxic effects, and significantly enhanced wound regeneration in a murine model (5% residual wound area after 18 days). These results establish rheological synergism as an important parameter, an index of the extent of polymer–protein interactions, that are responsible of the formulation performance.

## Graphical abstract

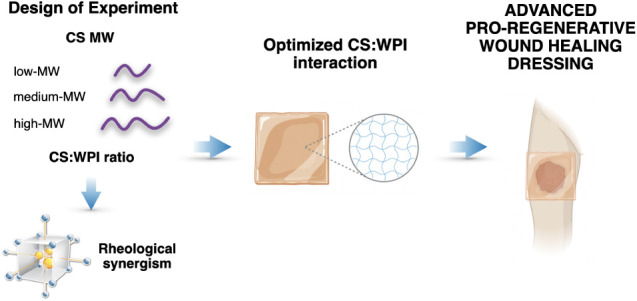

## Introduction

The skin, as the body largest and most complex organ, serves as the primary defense against external stimuli. It is responsible for several pivotal functions, including protection against pathogen invasion, mechanical stresses, and infections, as well as regulation of body temperature. In addition, the skin acts as an active immune organ, housing both the innate immune system cellular elements and the adaptive immune system.^1,2^ For these reasons, skin wounds, arising from various causes, represent a significant challenge for clinical management. Effective wound care is crucial to prevent infections, minimize scarring, and promote proper tissue regeneration.^3^ However, the complexity of wound healing process, which involves multiple overlapping biological processes, often complicates treatment and impairs healing.^4^

Traditional wound dressings, such as gauze and synthetic polymer-based materials, often fail to provide an optimal healing microenvironment. These materials lack key features such as antimicrobial activity, bioactive signaling, and controlled moisture management, resulting in prolonged healing times and an increased risk of complications.^5,6^ Therefore, there is a growing interest in the development of biobased dressings that incorporate natural polymers with enhanced biocompatibility, bioactivity, and mechanical performance thereby offering innovative solutions for wound care.^7^

The development of advanced wound dressings plays a crucial role in modern healthcare, particularly in enhancing wound healing and tissue regeneration. These materials can actively participate in the healing process by modulating inflammation, preventing bacterial infections, and promoting cell proliferation. Advanced wound dressings are available in various forms, including films, hydrogels, foams, and fibers, and can be further functionalized with bioactive agents such as antimicrobials, growth factors, and pro-regenerative molecules.^7–9^

Biomaterial-based wound dressings have been attracted considerable attention due to their ability to mimic the extracellular matrix, providing both structural support and biological cues essential for tissue regeneration.^10,11^ These materials, typically composed of natural polymers forming hydrogels, films, and nanofibers, offer advantageous properties, including controlled drug release, oxygen permeability, and biodegradability. In particular, hydrogel-like films can maintain a moist wound environment while enabling the exchange of bioactive molecules necessary for cellular migration and proliferation.^12,13^ Polymeric films also act as effective physical barriers against bacteria penetration and are widely used due to their biocompatibility and low immunogenicity. Moreover, they are noninvasive, easy to apply, allow gas exchange, and can be transparent, facilitating wound monitoring exudate assessment. Among the available fabrication techniques, solvent casting is one of the most commonly employed methods for producing polymeric films, owing to its simplicity and cost-effectiveness.^14,15^

Among the various biopolymers explored for wound healing applications, chitosan (CS) has received significant attention due to its unique physicochemical and biological properties.^16–18^ CS is a natural polysaccharide derived from chitin deacetylation and is characterized by excellent biocompatibility, biodegradability, antimicrobial activity, and hemostatic properties, making it a promising material for wound healing. Furthermore, its ability to stimulate fibroblast proliferation and accelerate re-epithelialization further strengthens its potential in wound care.^19–21^

Similarly, whey protein isolate (WPI), a byproduct of the dairy industry rich in bioactive peptides, possesses high water retention capacity and film-forming ability, which can further enhance the performance of wound dressings. WPI also contains essential amino acids that support cell adhesion and tissue repair, making it a valuable component in bioactive wound healing materials.^22–24^ However, WPI presents certain limitations, such as sensitivity to temperature, pH, and ionic strength; therefore, its incorporation into polymeric matrices can contribute to improved stability and functionality.^17^

The synergistic combination of CS and WPI, previously explored in the literature,^16–18^ is expected to result in materials with enhanced mechanical strength, moisture retention, and bioactivity, making them suitable candidates for wound dressing applications.^25^ Although several studies have investigated CS/WPI systems, these have been mainly focused on food packaging or edible films. To the best of our knowledge, their application as advanced skin wound healing devices has not been systematically explored.

Despite the increasing interest in CS/WPI-based systems, current studies mainly focus on material preparation and characterization for other applications. In the case of wound healing, a rational understanding of how polymer–protein interactions at the molecular level influence the final product functional performance is still lacking.

Given these premises, the aim of the present work is to evaluate the influence of polymer–protein interactions on the resulting film mechanical and functional properties.

To this end, CS-WPI mixtures prepared with different CS MWs and different CS:WPI ratios were characterized in terms of rheological behavior. A Design of Experiments (DoE) approach was employed to investigate the effect of the influence of the CS MW and of CS-WPI weight ratio on rheological synergism, which is indicative of the extent of interaction between components.^18^ Subsequently, films based on the most promising mixtures were produced via solvent casting and systematically evaluated for their mechanical properties, absorption capacity, wettability, and biodegradability. In addition, in vitro biocompatibility, antimicrobial activity tests, cell proliferation assays, as well as in vivo experiments on a murine model were conducted to assess their effectiveness in preventing infections and promoting skin wound regeneration.

Importantly, this study demonstrates that rheological synergism can be used to guide the rational design of polymer–protein-based biomaterials, bridging formulation variables and final functional performance.

## Experimental Section

### Materials

Low molecular weight chitosan (l-CS), 50–190 kDa, degree of deacetylation 76%, medium molecular weight chitosan (m-CS), 190–310 kDa, degree of deacetylation 85%, and high molecular weight chitosan (h-CS), 310–375 kDa, degree of deacetylation 75%, were purchased from Sigma-Aldrich (Italy). Whey protein isolate (WPI) were provided by Milei GmbH (Germany).

### Methods

#### Preparation of Cs:WPI Films

WPI was dissolved in Milli-Q water under magnetic stirring at 150 rpm to obtain a 4% w/v solution. Subsequently, the pH of the solution was adjusted to 7 by dropwise addition of 1 M NaOH, followed by denaturation at 70 °C for 20 min.^18^ Finally, the solution was cooled at room temperature. CS solution was prepared as 4% w/v solution in 0.5 M acetic acid under magnetic stirring at 200 rpm. Afterward, the CS solution was centrifugated at 8000 rpm for 10 min at 20 °C (Centrifuge Hermle Z326 K, Germany), to remove insoluble impurities originating from CS production process. Then the pH was adjusted to 4.5 by dropwise addition of 0.1 M NaOH. The pH adjustments were performed to promote interactions between negatively charged proteins and positively charged chitosan.

The final mixtures were prepared by combining WPI and CS solutions maintaining a constant solid content of 4%, but varying the volume ratios, as shown in Table 1. In addition, to obtain the final film-forming solution, glycerol (GLY), was added as plasticizing agent at 60% w/w relative to the dry weight of the proteins.

**Table 1:** Quali-/Quantitative Composition of CS:WPI Mixtures

Mixtures Volume Ratio	WPI (% w/w)	CS (% w/w)
3:1	3	1
2:2	2	2
1:3	1	3

As for film preparation, the solvent casting method was employed: 3.3 mL of the film-forming solutions were poured into silicon molds (Ø = 6 cm), followed by drying in an oven at 70 °C for 2 h. This preparation method was repeated for the three molecular weights (MW) of CS.

#### Characterization of Cs:WPI Mixtures

##### Rheological Analysis

Rheological analysis of the CS:WPI different mixtures was performed using a rotational rheometer (MCR 102, Anton Paar, Turin, Italy) equipped with a cone plate geometry (CP50–1, diameter = 50 mm; angle = 1°) as measuring system. Viscosity measurements were carried out at increasing shear rates from 10 to 300 s^–1^ at 20 °C with a gap of 0.101 mm. Three replicates were performed for each sample.

After the analysis, the normalized rheological synergism parameter (Δ $\frac{\eta }{\eta }$ ) was calculated at all shear rates to investigate the interaction between the two components mixed in different ratios, as reported in Equation 1:^26^

1 $$\Delta \frac{\eta }{\eta }=\frac{\eta_{CS:WPI}-\left[\eta \left(CS\right)+\eta \left(WPI\right)\right]}{\left[\eta \left(CS\right)+\eta \left(WPI\right)\right]}$$

Where:

- *η_CS_*_:_ *_WPI_* = viscosity of each CS:WPI mixture
- *η*(*CS*) + *η*(*WPI*) = theoretical value calculated as the sum of the individual viscosities of CS and WPI solutions at the same concentrations as in the respective mixture.

##### Design of Experiments

A Design of Experiments (DoE) was conducted to statistically evaluate the contribution of CS MW and CS-WPI weight ratio on rheological synergism parameter $\frac{\Delta \eta }{\eta }$ , index of the extent of interactions between CS and WPI. A full factorial design was selected, including all possible combinations of the considered factors and levels. Specifically, the experimental model included 2 factors (CS MW and CS:WPI ratio), each studied at 3 different levels: high (+1), medium (0), and low (−1), resulting in a 3^k^ factorial design (k = 2). In detail:

- CS MW: low (−1), medium (0), high (+1)
- CS:WPI ratio: 1:3 (−1), 2:2 (0), 3:1 (+1)

The response variable selected for the DoE was $\frac{\Delta \eta }{\eta }$ (calculated at 100 s^–1^) with three replicates for each experiment. Chemometric Agile Tool software was employed to generate the statistical model that best fits the experimental data.

#### Characterization of CS:WPI Films

##### Mechanical Properties Analysis: Static and Dynamic Properties

CS:WPI films static mechanical properties were assessed using a TA.XT plus Texture Analyzer (Stable Micro Systems, United Kingdom) equipped with a 5 kg load cell. Before analysis, film thickness was measured by means of a Sicutool 3955G-50 (Italy) apparatus. Samples (1 × 3 cm^2^) were clamped with A/TG tensile grips, setting a distance of 1 cm between the grips. The process parameters related to the speed during analyses were: *pretest speed* = 1.00 mm/sec; *test speed* = 0.5 mm/sec; *post-test speed* = 0,5 mm/sec. The following parameters were calculated: maximum tensile strength (TS, MPa) and elongation at break % (EB %). For TS, the data obtained as force (N) values were normalized for the cross-sectional area, calculated by multiplying the film thickness (about 0.20 mm) by its width (10 mm); six replicates were carried out for each sample. Eight replicates were carried out for each sample.

Additionally, Dynamic Mechanical Analysis (DMA; MCR 702e MultiDrive, Anton Paar, Austria) was performed in torsion mode, using SRF units as clamp systems, to assess films dynamic mechanical properties. First, an amplitude strain sweep test was performed at a fixed frequency of 1 Hz over a strain range of 0.0001–1% to determine the linear viscoelastic region (LVR) of the films. Once the strain % value corresponding to $\frac{2}{3}$ of the LVR was identified, this value was used for subsequent frequency strain sweep test. These tests were conducted at a constant strain within the LVR while increasing the frequencies (Hz) over a range of 1–20 Hz. Three replicates were performed for each sample.

##### Hydration Properties Analysis

The hydration properties of the films were evaluated in phosphate-buffered saline (PBS; VWR Chemicals, LLC, USA). The wells of a 12-well plate were covered with a nonwoven membrane, and each well was topped with a dialysis membrane, previously conditioned at 80 °C in Milli-Q water for 15 min, on which the sample was placed. The swelling ratio and, consequently, the PBS absorption capacity of each sample were calculated using the following formula:

2 $$Swelling\;ratio\%=\frac{W_{f}-W_{i}}{W_{i}}x100$$

Where:

- *W_i_* is the weight of the sample measured at time 0
- *W_f_* is the weight of each film corresponding to the final weight measured at each time point considered (1, 3, 6, and 24 h).

Four replicates were analyzed for each sample.

Moreover, Water Contact Angle was measured using a DMe-211 Plus Contact Angle Meter (Kyowa Interface Science Co Ltd., Japan) according to the θ2 method. For the test, a drop (5 μL) of deionized water was deposited onto the surface of films, and the contact angle was measured over time using FAMAS Dropmaster Software. The initial measurement time was set at 100 ms, and a recording time of 10,000 ms for a sequence of 30 scans was used. Three replicates were performed for each sample.

##### Viscoelastic Properties Analysis

Stress sweep measurements were carried out at 32 °C on films hydrated for 24 h using a rotational rheometer (Modular Compact Rheometer 102, Anton Paar s.r.l., Austria), equipped with a plate–plate system (PP25; Ø = 25 mm). A constant frequency of 0.1 Hz was applied while shear strain was increased from 0.1 to 20% in order to determine the linear viscoelastic region (LVR). The strain corresponding to 23 of the linear portion of the LVR was identified as the maximum strain applicable to the film for subsequent oscillation measurements without disrupting their internal structure. Accordingly, oscillatory frequency sweep tests were carried out at a constant temperature (32 °C) over a frequency range of 1–10 Hz. Two replicates were performed for each film. The storage (elastic) modulus (G′) and the loss (viscous) modulus (G″) were recorded as a function of frequency. The loss factor (tgδ) was then calculated as the ratio G‴/G″ to assess film viscoelastic behavior after hydration. Three replicates were performed for each sample.

#### In Vitro and In Vivo Assays

##### In Vitro Biodegradation Assay

A biodegradation test was performed on films consisting of WPI and CS in the ratio identified as the most promising through the experimental design (CS:WPI 1:3) using all three CS MWs. Two degradation media were considered: PBS and PBS + H_2_O_2_ 500 μM (a medium simulating wound bed conditions).^27^ The response parameters considered were the residual mass (%) determined by gravimetric assessment and spectrophotometric analysis of the supernatants.

Briefly, films (about 20 mg) were placed into Eppendorf tubes containing 5 mL of the degradation medium and maintained at 32 °C in a heating/shaking bath (FALC Instruments, Italy). Gravimetric measurements were performed at predetermined time points of 1, 3, and 7 days.

3 $$Residual\;mass\%=\frac{Wf}{Wi}\times 100$$

where:

W_f_ = final weight, namely the weight of the films after hydration at prefixed time points;

W_i_ = initial dry weight.

In addition, 3 mL of the supernatant were recovered at each time point and analyzed by UV–Vis spectrophotometry (PerkinElmer Lambda 25 UV/vis). Absorbance (A) was measured at 280 nm, corresponding to the maximum absorption wavelength of proteinogenic amino acids. Protein concentrations were calculated using a calibration curve previously generated with the same film-forming solution used for film preparation. Results were expressed as the amount of protein released from the films at each time point. Three replicates were performed for each sample.

##### In Vitro Antimicrobial Properties Analysis

The antimicrobial activity of CS:WPI films was evaluated using circular samples obtained with a 4 mm biopsy punch and placed into individual wells of a sterile 96-well plate containing 100 μL of Luria–Bertani (LB) medium per well. The plates were incubated for 24, 48, or 72h at RT. *Staphylococcus aureus* BH1CC (*S. aureus* *)* and *Pseudomonas aeruginosa* PAO1 (*P. aeruginosa* *)* strains, previously cultured overnight in LB medium, were diluted in fresh medium and grown to the exponential phase (OD_600_ = 0.5–0.8). Approximately 5 × 10^5^ cells were inoculated into each well, in the presence or absence of the films, and incubated overnight at 37 °C. Following incubation, bacterial suspensions were transferred to sterile microplates, and optical density at 600 nm (OD_600_) was measured using a microplate reader to assess bacterial growth. Results were expressed as the percentage of bacterial growth relative to the control cultures incubated without films.

##### In Vitro Indirect Cytocompatibility, Proliferation, and Wound Healing Assay

The cytotoxicity of the films was assessed by an indirect viability test on normal human dermal fibroblasts (NHDF). NHDF were cultured in polystyrene flasks (Cellstar tissue culture flasks, Grainer Bio-One, Italy) in a complete culture medium (CM), consisting of DMEM-HG, supplemented with 10% v/v of fetal bovine serum (FBS), previously inactivated in a water bath at 56 °C for 30 min, and 1% of an antibiotic/antimycotic solution based on penicillin/streptomycin/amphotericin 100X. Cells were maintained in an incubator (CO_2_ Incubator, PBI International, Italy) with a controlled atmosphere of 37 °C, with relative humidity of 95%, in the presence of 5% CO_2_.

The samples were sterilized by UV rays with two cycles of 20 min each. Each sample was placed in a tube (Biosigma, Italy) containing 600 μL of CM, then placed in the incubator for 24 h at 37 °C and 5% CO_2_. Meanwhile, NHDF were seeded in a 96-well Multiwell (Corning 96 Well TC-Treated Microplates) at a density of 100,000 cells/cm^2^ and left in the incubator at 37 °C for 24 h. Subsequently, the CM was removed from the wells and replaced with 200 μL of conditioned CM from the samples (extracts). After 24 h, a MTT test was performed to evaluate the cytotoxic effect exerted by the films extracts on cell viability. Briefly, films extracts were removed from each well, and after PBS (100 μL) washing, 150 μL of 0.83 μg/mL MTT in DMEM without phenol red were added. The plate was placed in the incubator for 3 h, to promote the development of the colorimetric reaction. After MTT removal, 100 μL of DMSO were added to each well in order to promote complete dissolution of the formed formazan salts, producing a characteristic violet color. The absorbance of each well was measured spectrophotometrically by microplate reader (FLUOstar Omega Microplate Reader, BMG Lab Tech, Germany) at a wavelength of 570 and 690 nm (reference wavelength), after 60 s of 100 rpm agitation.

In addition, also a proliferation assay was carried out. Cells and samples were treated in the same ways as for the cytocompatibility assay, but in this case, they were cocultured (cell density of 50,000 cells/cm^2^) and a MTT test was performed after 24 h of incubation at 37 °C and 5% CO_2_. Six replicates were carried out for each sample.

Finally, a live wound healing assay was performed to assess the effect of films extracts on the migratory capacity of NHDF. Briefly, NHDF were seeded into Ibidi culture inserts (2-well, 24-well plate format; Ibidi GmbH, Gräfelfing, Germany), each consisting of two chambers (growth area: 0.22 cm^2^) separated by a 500 ± 50 μm cell-free gap, mimicking a wound site. Cells were seeded at a density of 10,000 cells per chamber and cultured for 24 h to allow adhesion and confluence. The inserts were then carefully removed to initiate the wound healing process, and films extracts (treated as for cytocompatibility and proliferation assays) were added to each well. The plates were maintained incubated at 37 °C with 5% CO_2_, and images of the wound area were acquired every 15 min over a 72 h period using a Leica TCS SP2 Confocal Laser Scanning Microscope (Leica Microsystems, Milan, Italy). Wound closure was quantified by measuring the residual gap area at each time point with ImageJ 2.0 software, and expressed as residual gap area (%) relative to the initial gap area, taken as 100%. Each condition was tested in six replicates to ensure data reliability.

4 $$Residual\;gap\;area\;\left(\%\right)=\frac{A_{t}}{A_{0}}\times 100$$

where:

*A*_t_ = area of the gap at each time point.

*A*_0_ = initial area of the gap at day 0.

##### In Vivo Experiments on Murine Animal Model

All animal procedures were conducted in compliance with the international ethical guidelines for animal care (European Communities Council Directive 2010/63/EU) and were approved by the Italian Ministry of Health (D.L. 116/92), the Local Institutional Ethics Committee of the University of Pavia, and the Istituto Superiore di Sanità.

Six male Wistar rats (200–250 g; Envigo RMS S.r.l.) were anesthetized with equitensine (3 mL/kg; 39 mM pentobarbital, 256 mM chloral hydrate, 86 mM MgSO_4_, 10% v/v ethanol, and 39.6% v/v propylene glycol). The dorsal region of each animal was shaved and three circular full-thickness burns (4 mm in diameter) were created using a preheated aluminum rod (105 °C for 40 s). After 24 h, the resulting blisters were excised with a 4 mm biopsy punch to obtain standardized full-thickness lesions.

Film samples were prepared under sterile conditions in a laminar flow hood, cut with a 4 mm biopsy punch, and sterilized by 24 h UV exposure prior to application. Each lesion received either a sterilized film or 20 μL of saline solution (negative control). Wounds were then covered with sterile gauze and protected using a surgical stretch bandage (Safety, Italy).

Animals were monitored daily by veterinary staff throughout the study. Digital images of the wounds were taken at days 0, 4, 7, 11, 14, and 18. Residual wound area (%) was quantified using ImageJ software according to the equation:

5 $$Residual\;wound\;area\;\left(\%\right)=\frac{A_{t}}{A_{0}}\times 100$$

where:

*A*_t_ = area of the wound area at each time point (days 4, 7, 11 and 18).

*A*_0_ = initial area of the wound at day 0.

At day 18, full-thickness biopsies were collected from each lesion as well as from intact skin (reference). Samples were fixed in 4% w/v neutral buffered paraformaldehyde for 48 h, dehydrated through graded ethanol series, cleared in xylene, and embedded in paraffin. Sections were stained with hematoxylin and eosin (H&E) for general histology or picrosirius red (PSR) for collagen visualization. Following deparaffinization and rehydration, sections were counterstained with Weigert’s hematoxylin, dehydrated, cleared in xylene, and mounted using DPX medium. Histological evaluation was performed using a Carl Zeiss Axiophot light microscope equipped with circular polarizing filters. Images were captured using a Nikon DS-Fi2 5-megapixel CCD digital camera.

#### Statistical Analysis

Where applicable, data were subjected to statistical analysis by means of Astatsa statistical calculator; one-way analysis of variance (ANOVA) was applied, followed by Scheffé post hoc comparisons. A significance level of *p* < 0.05 was considered statistically significant.

## Results and Discussion

A growing trend in wound healing research is the use of biopolymers derived from natural and renewable sources to fabricate innovative dressings that are highly biocompatible and biomimetic, thereby promoting cell proliferation and tissue repair. Among them, CS is extensively investigated and commonly applied in the form of films, hydrogels, fibers, and scaffolds.^28–30^ WPI also represents a promising candidate due to its biodegradability, bioactivity, and excellent film-forming ability, mainly attributed to intermolecular disulfide bond formation upon protein denaturation.^9^

In this work, CS:WPI films were prepared at different weight ratios (1:3, 2:2, and 3:1) using CS at three molecular weights (low, medium, high). The rheological interaction between CS and WPI was evaluated through the calculation of the normalized rheological synergism parameter $\left(\frac{\Delta \eta }{\eta }\right)$ ; the rheological synergism values calculated at 100 s^–1^ are reported in Figure 1. As can be observed in the graph, the highest values of $\frac{\Delta \eta }{\eta }$ were consistently obtained for the 1:3 CS:WPI mixtures, independently of CS MW, suggesting that lower CS content favors intermolecular interactions between the two components. The same trend was observed at all the shear rates considered. Notably, the combination with m-CS exhibited the strongest synergistic effect, indicating the highest interaction between the two components. It is worth noting that m-CS is characterized by a higher deacetylation degree (DD) (85%) compared to l-CS and h-CS (∼75–76%). This difference in DD involves a higher amount of protonated amino groups in m-CS, which should strengthen the electrostatic interactions with the negatively charged groups of denatured WPI.^31^ However, the main goal of this work was not to isolate the effect of a single structural parameter, but rather to identify the most suitable chitosan grade for wound dressing development within a formulation-driven framework.

**Figure 1:**
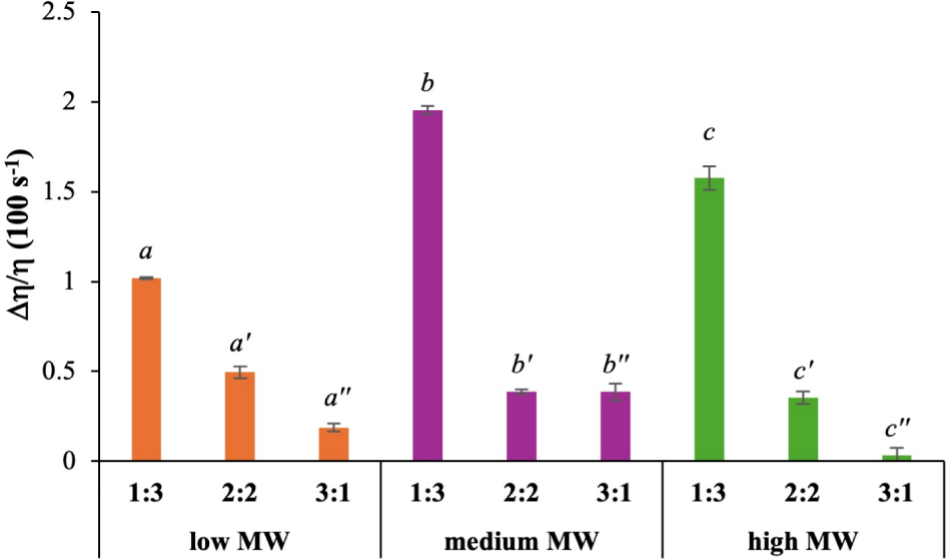
Normalized rheological synergism parameter $\left(\frac{\Delta \eta }{\eta }\right)$ calculated at 100 s^–1^ for CS:WPI mixtures prepared at different weight ratios and using CS of different MW (mean values ± SD; *n* = 3); one-way ANOVA followed by Scheffé post hoc test, *p* < 0.05: a vs a′, a‴, b, c; a″ vs a‴, b″, c′; a‴ vs b‴, c‴; b vs b″, b‴, c; b⁗ vs c‴; c vs c′, c‴; c″ vs c″.

A full factorial design (3^2^), reported in Table 2, was applied to statistically evaluate the influence of CS MW and CS:WPI ratio on rheological synergism, selected as the response variable indicating the occurrence of an interaction product between the two components. The response surface contour plot shown in Figure 2B provides a comprehensive 2D visualization of the predicted outcome (Δη/η) as a function of CS:WPI ratio (factor A) and CS MW (factor B).

**Figure 2:**
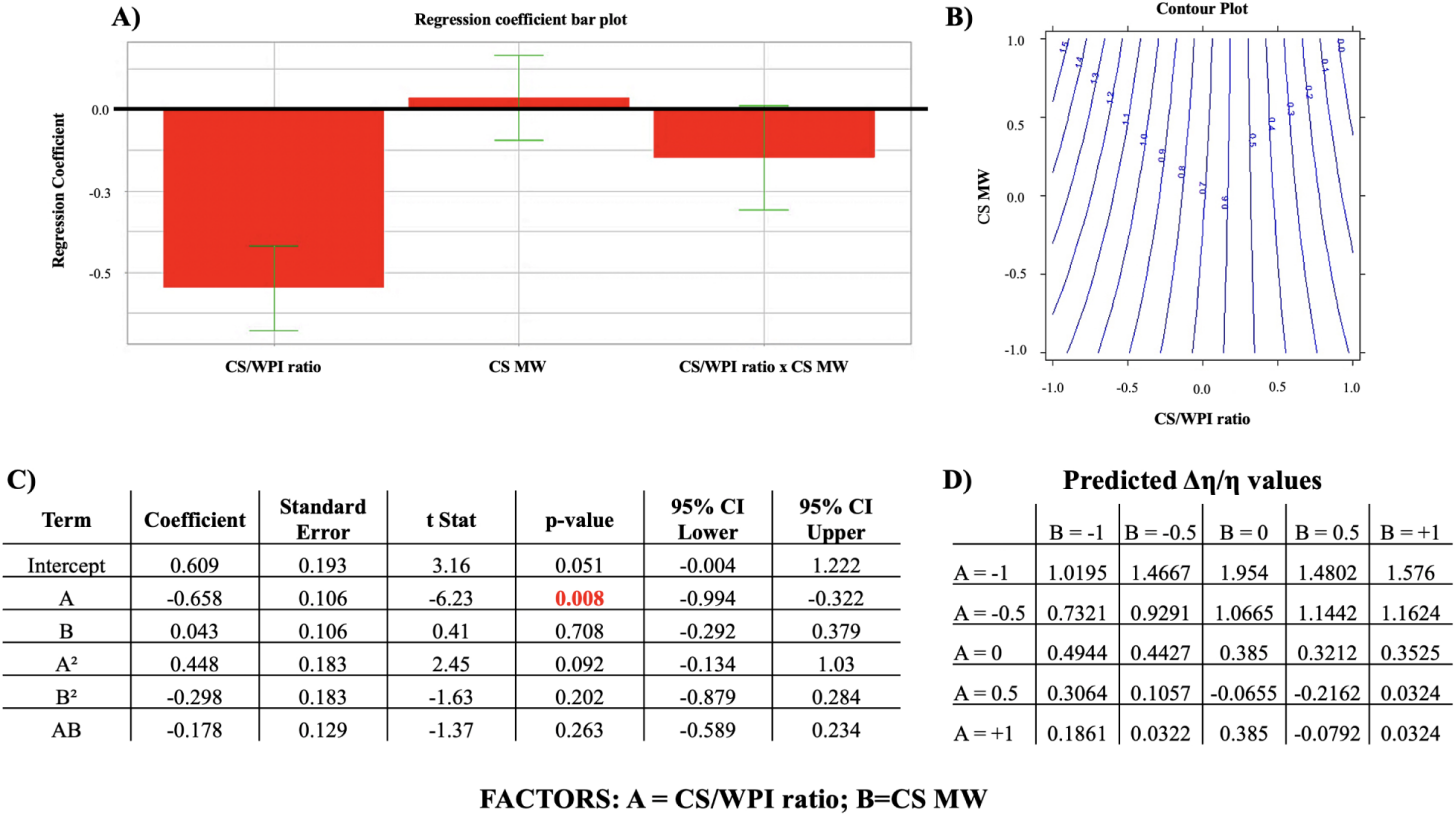
Results of the 3^2^ full factorial design showing the effect of CS MW and CS:WPI ratio on the normalized rheological synergism parameter $\left(\frac{\Delta \eta }{\eta }\right)$ , with a 95% confidence interval: A) Regression coefficient plot showing the estimated effects of the investigated factors and their interaction; error bars represent 95% confidence intervals. B) Contour plot showing the predicted Δη/η values as a function of CS:WPI ratio and CS MW. C) Regression coefficients, standard errors, t statistics (t Stat), p-values, and 95% confidence intervals (CI) of the fitted quadratic model. D) Predicted Δη/η values calculated from the fitted model for the investigated factor combinations.

**Table 2:** Full Factorial Design 3^2^

		**Variables**		**Levels**	
**Experiments**	**Samples**	CS:WPI ratio	**CS MW**	CS:WPI ratio	**CS MW**
**1**	l-CS1_WPI3	1:3	low	–1	–1
**2**	l-CS2_WPI2	2:2	low	0	–1
**3**	l-CS3_WPI1	3:1	low	+1	–1
**4**	m-CS1_WPI3	1:3	medium	–1	0
**5**	m-CS2_WPI2	2:2	medium	0	0
**6**	m-CS3_WPI1	3:1	medium	+1	0
**7**	h-CS1_WPI3	1:3	high	–1	+1
**8**	h-CS2_WPI2	2:2	high	0	+1
**9**	h-CS3_WPI1	3:1	high	+1	+1

The model demonstrates a high predictive capability (R^2^ = 0.94) as reported in Figure 2C, confirming both the reliability of the experimental design and the robustness of the fitted surface.

The canonical quadratic model, y = b_0_ + b_1_ × A + b_2_ × B + b_12_ × A × B + b_11_ × A^2^ + b_12_ × B^2^, was identified as the best-fitting model to describe the observed response. The model equation computed for the selected response is

6 $$\Delta \eta /\eta =0.609-0.658\times A+0.043\times B-0.178\times A\times B+0.448\times A^{2}-0.298\times B^{2}$$

The bar plot shown in Figure 2A reveals that the CS:WPI ratio exerts the most significant influence on the response (p = 0.008), with higher ratios leading to a marked decrease in Δη/η. The confidence intervals reported in the coefficient plot indicate the uncertainty associated with each estimated model term. Among the investigated factors, only the CS:WPI ratio showed a statistically significant effect on rheological synergism, as confirmed by its confidence interval not crossing zero (y = 0 black line) and by the corresponding p-value (p = 0.008), highlighted in Figure 2C. This trend is further supported by the presence of a quadratic effect, indicating a nonlinear relationship between the CS:WPI ratio and the response variable considered. Conversely, the effect of CS MW and its interaction with CS:WPI ratio appear to be less pronounced within the studied range, in fact they did not have a statistically significant effect on the response variable (*p* > 0.2). To further validate the predictive ability of the quadratic model, the equation was used to compute Δη/η values for all combinations of CS:WPI ratio and CS MW, as displayed in Figure 2D.

These results indicate that the CS:WPI ratio is the key parameter for tuning the rheological properties of the mixtures, while variations in CS MW have a comparatively minor impact. The model can be used to predict the response for any combination of the studied factors, facilitating the optimization of formulation parameters. Therefore, based on these findings, the 1:3 CS:WPI ratio – the lowest weight ratio considered – was selected for further investigations. Overall, these findings suggest that rheological synergism can serve as an indicator of polymer–protein interaction strength and, consequently, of the final film performance. This approach provides a valuable tool for guiding the rational design of composite biomaterials beyond empirical trial-and-error strategies.

Figure 3 shows the values of $\frac{\Delta \eta }{\eta }$ of such mixtures calculated in a shear rates range between 10 s^–1^ and 300 s^–1^. In particular, it can be observed how the m-CS:WPI mixture is characterized by a higher rheological synergism profile compared to that of the l-CS:WPI and h-CS:WPI mixtures in the whole range considered. Therefore, even though CS MW did not have a statistically significant impact on the rheological synergism considering the quadratic model built for data analysis, mixtures composed by m-CS seemed to allow the best interaction with WPI. This result suggests that the balance between chain flexibility and intermolecular bonding is optimal at this MW. These findings are in line with previous reports highlighting the importance of polymer–protein interactions in tuning the properties of composite biomaterials.^32,33^

**Figure 3:**
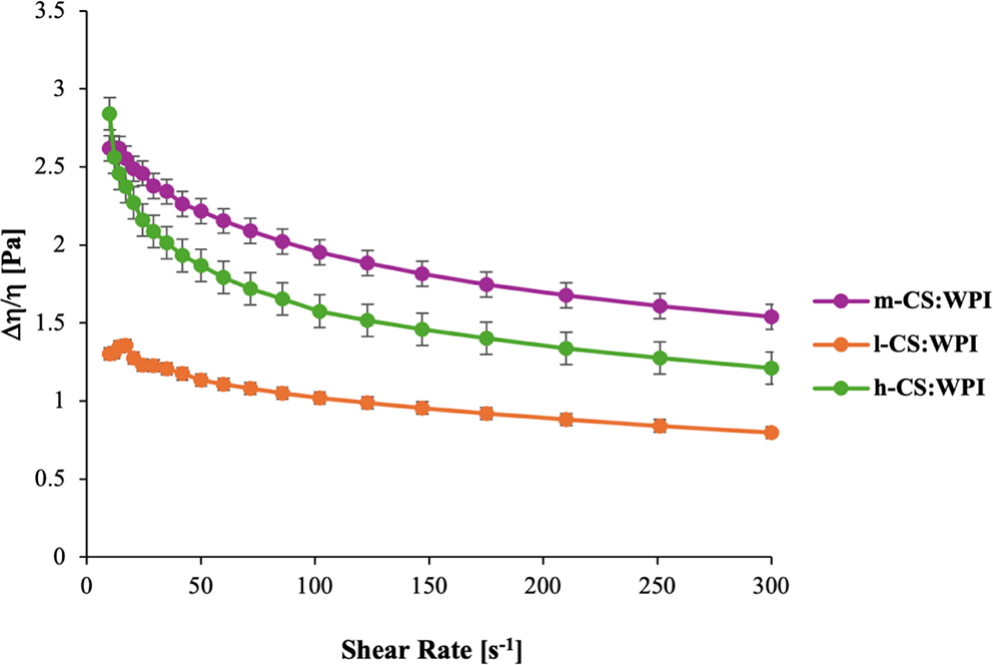
Values of the normalized rheological synergism parameter (Δη/η), calculated in a shear rates range between 10 s^–1^ and 300 s^–1^, of CS:WPI mixtures, prepared in a 1:3 weight ratio, using CS at different MW (mean values ± SD; *n* = 3).

CS:WPI mixtures were then added with GLY, used as a plasticizing agent, to obtain three film-forming solutions (l-CS:WPI S, m-CS:WPI S and h-CS:WPI S). Glycerol is widely recognized as an effective plasticizer for both CS and WPI-based films, as it significantly improves their flexibility and workability by reducing intermolecular forces and increasing polymer chain mobility.^34,35^

Figure 4 shows the viscosity (a) and flow (b) curves of the solutions: all samples are characterized by a pseudoplastic behavior (Figure 4A) with a decreasing viscosity trend as CS MW decreases. As expected, shear stress values increase with increasing CS MW over the entire range of shear rates considered (Figure 4B).

**Figure 4:**
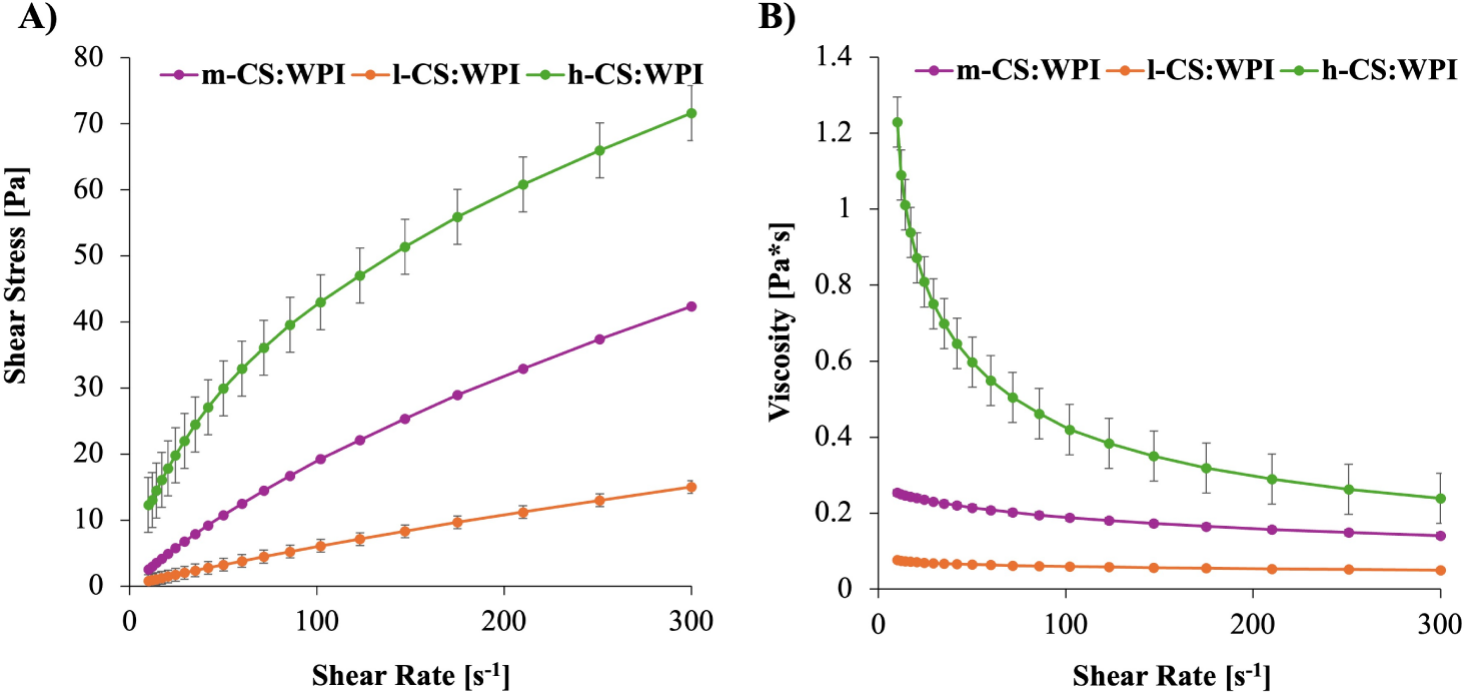
Flow (a) and viscosity (b) curves of l-CS:WPI S, m-CS:WPI S and h-CS:WPI S solutions from 10 s^–1^ to 300 s^–1^ (mean values ± SD; *n* = 3).

The CS:WPI mixtures added with GLY were used to obtain films prepared via solvent casting technique. The solvent casting technique is widely used for the preparation of polymeric films due to its simplicity, low cost, and ability to produce homogeneous films with controlled thickness.^36^ The resulting films were first characterized in terms of their mechanical properties under both statical and dynamical conditions. The results of the statical mechanical test are reported in Figure 5, in terms of A) TS and B) EB %. Mechanical testing revealed that m-MW CS film exhibited the highest TS, which was statistically higher than that of l-CS and h-CS based films. This behavior could be attributed to enhanced network formation between m-CS and WPI, as also supported by rheological data. Regarding EB % it was observed that films composed by l-CS and m-CS were characterized by similar values, higher than those observed for h-CS ones. The h-CS:WPI film, derived from an excessively viscous solution, exhibited the poorest mechanical performance in terms of both TS and EB %. It should be noted that the very high viscosity of the h-CS:WPI solution made it difficult to pour into the molds prior to film preparation, which may have adversely affected the uniformity and, consequently, the final properties of the films.

**Figure 5:**
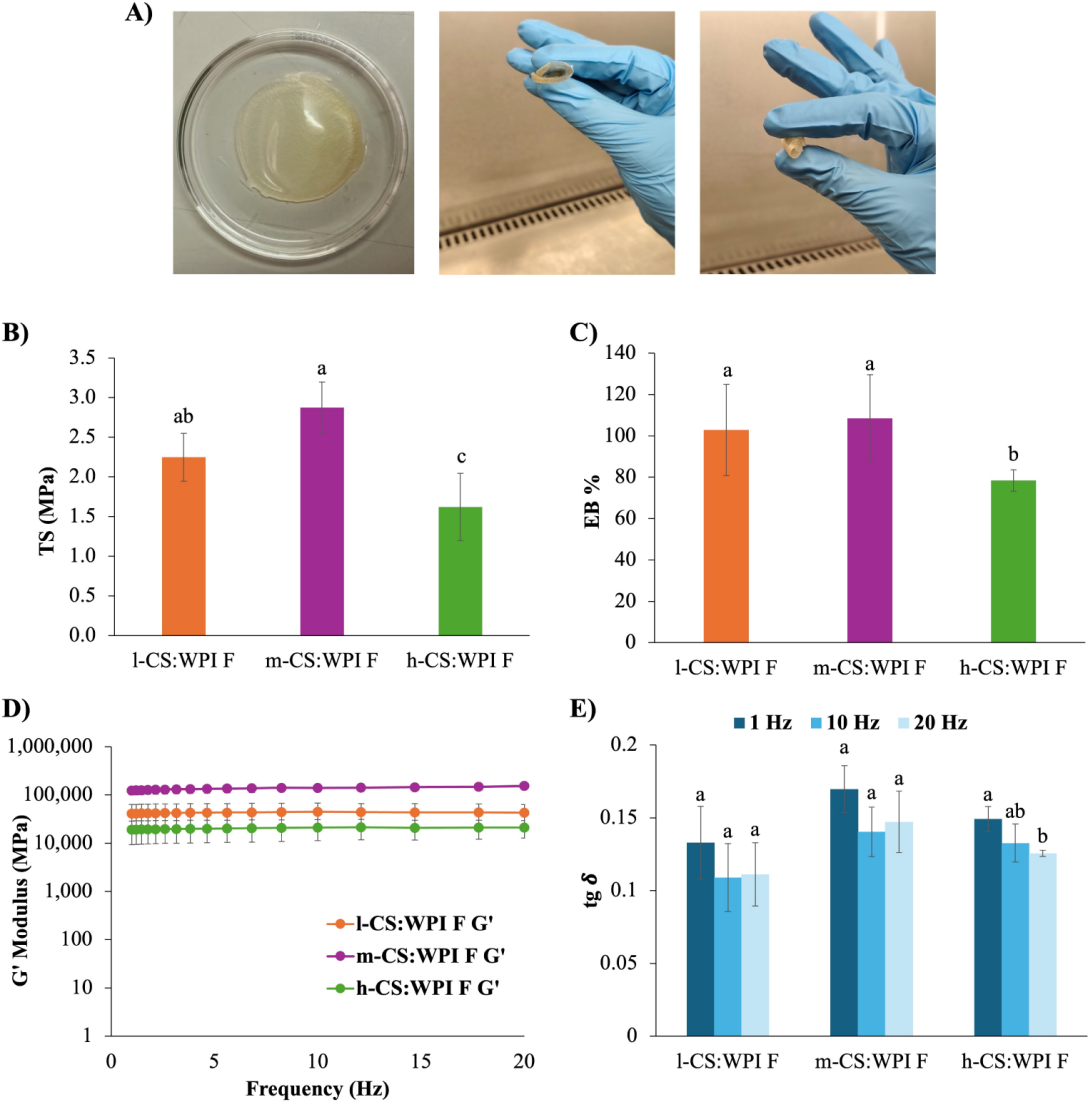
A) Photographs of representative m-CS:WPI F showing their macroscopic appearance after solvent casting in a Petri dish (left), upon folding (middle) and rolling (right) to show its handling and flexibility. B) Maximum Tensile strength (TS, MPa) and C) Elongation at break % (EB %) values of the films (mean values ± SD; *n* = 8). D) G′ modulus (MPa) of the CS:WPI films at different CS MW in a 1–20 Hz frequency (Hz) range (mean values ± SD; *n* = 3; E) loss factor (tg δ) calculated at 1, 10, and 20 Hz for all the CS:WPI films (mean values ± SD; *n* = 3). Statistical analysis was performed by one-way ANOVA followed by Scheffé post hoc test. Different letters indicate statistically significant differences among samples within the same experimental condition or frequency (*p* < 0.05), whereas shared letters indicate no significant differences.

Regarding dynamical mechanical properties, frequency sweep tests were performed by DMA in torsional mode using a shear strain of 0.1079%; this analysis further highlighted significant differences in the viscoelastic behavior of CS:WPI films depending on CS MW. As shown in Figure 5C, all samples are characterized by high G′ values, ranging from 10,000 to 100,000 MPa over the entire frequency range investigated. Moreover, regardless of CS MW, the G′ values are nearly independent of frequency; this behavior is characteristic of well-structured polymer networks and is commonly observed in materials characterized by a stable, cross-linked network with limited chain mobility, capable of store mechanical energy efficiently and recover its original structure after deformation.^37^ This interpretation is further supported by the tan δ (G‴/G″) values, which remain well below 1 for all samples and frequencies considered (see Figure 5D). Low tan δ values confirm the predominance of elastic behavior and the solid-like nature of the films, in agreement with recent literature on biopolymer-based films and hydrogels.^38,39^

Notably, the m-CS:WPI film exhibited the highest G′ values, followed by the h-CS:WPI and l-CS:WPI films. This trend suggests that the use of m-CS promotes the formation of a more robust and interconnected polymer network, likely due to an optimal balance between chain entanglement and molecular mobility, resulting in enhanced elastic properties.

Figure 6A shows the hydration properties of the films under study, expressed as swelling ratio % after 1, 3, 6, and 24 h of contact with PBS, used to simulate wound exudate. All films showed a time-dependent increase in swelling ratio, and, as expected, the swelling ratio was inversely related to CS MW, with l-CS:WPI films exhibiting the highest water uptake. This can be attributed to the fact that a higher polymer MW leads to greater chain entanglement, resulting in reduced PBS penetration into the three-dimensional polymeric network. It should be underlined that all films are able to absorb a remarkable amount of the aqueous medium (>150%) indicating their ability to control wound humidity. Proper moister content is crucial, if excessive could cause maceration of the surrounding tissue, impairing the healing process.^40^

**Figure 6:**
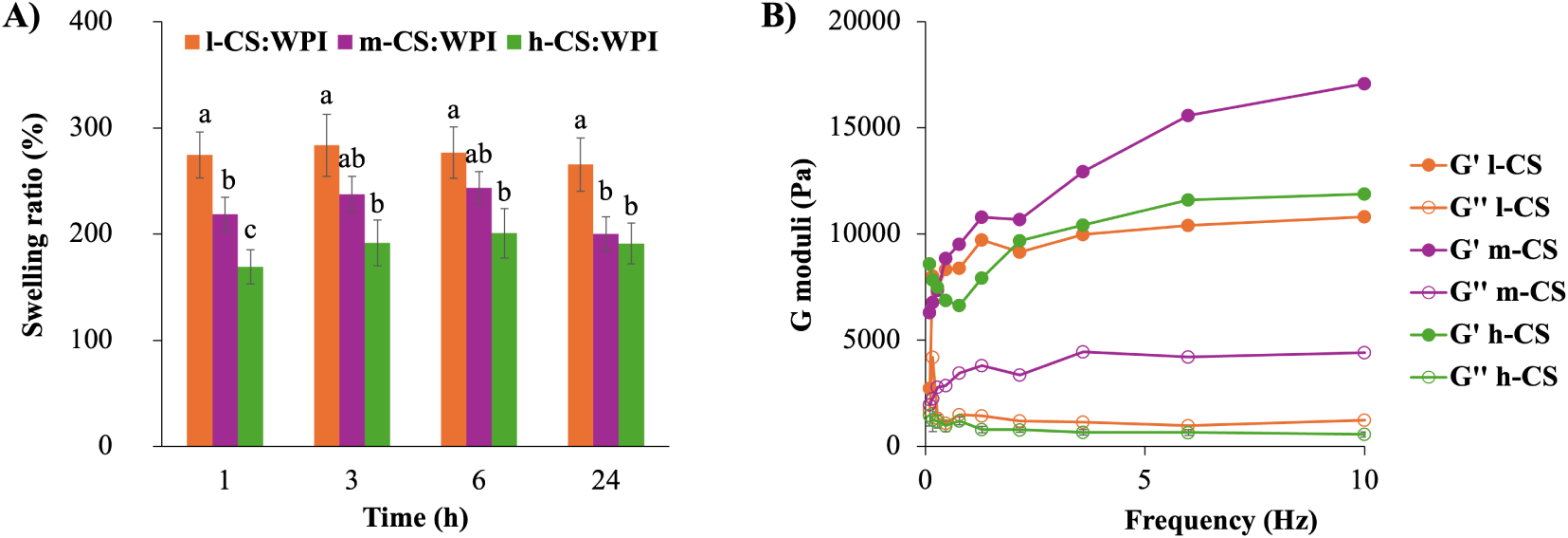
Hydration and viscoelastic behavior of CS:WPI films. A) Swelling ratio % of CS:WPI films after 1, 3, 6, and 24 h of immersion in PBS. Data are expressed as mean ± SD (*n* = 3). Statistical analysis was performed by one-way ANOVA followed by Scheffé post hoc test. Different letters indicate statistically significant differences among samples at the same time point (*p* < 0.05), whereas shared letters indicate no significant differences. B) Comparison between the storage modulus (G′) and loss modulus (G″) of the investigated CS:WPI films after 24 h of hydration in PBS (mean values; *n* = 3; CV% < 15%).

The viscoelastic properties of the hydrated films were evaluated after 24 h of hydration and are reported in terms of storage (G′) and loss (G″) moduli over the frequency range 1–10 Hz. In all cases, G′ was higher than G″ across the entire frequency range, indicating the predominance of elastic over viscous behavior. Notably, m-CS:WPI film retained the highest G′ profile even in the hydrated state, suggesting enhanced elasticity. It is also worth noting that the hydrated films exhibited markedly lower G′ values (about 8,000–10,000 Pa at 1 Hz) compared to their dry counterparts (as previously discussed). This reduction is due to the absorption of aqueous fluid upon hydration, leading to the formation of a hydrogel-like structure in which water becomes an integral component of the three-dimensional polymeric network. According to the literature, hydrogels intended for wound dressing applications commonly exhibit a storage modulus (G′) in the range of 100 to 10,000 Pa, ensuring an appropriate balance between mechanical stability, flexibility, and adaptability to the wound site.^41,42^

The elastic response of the hydrated film is particularly relevant for its protective role when applied to a wound. A film endowed with elastic behavior can absorb mechanical stresses by deforming and subsequently recover its original shape once the stress is removed, thereby contributing to wound protection.^43,44^

Wettability tests were then performed, and the results are reported in Figure 7A and B as representative images captured at 10, 100, and 300 s and as contact angle values measured over time, respectively. As observed, all samples exhibited contact angle values below 90°, confirming their hydrophilic nature. Notably, the CS:WPI F containing m-MW CS showed the lowest contact angle, indicating superior wettability compared to the other films.

**Figure 7:**
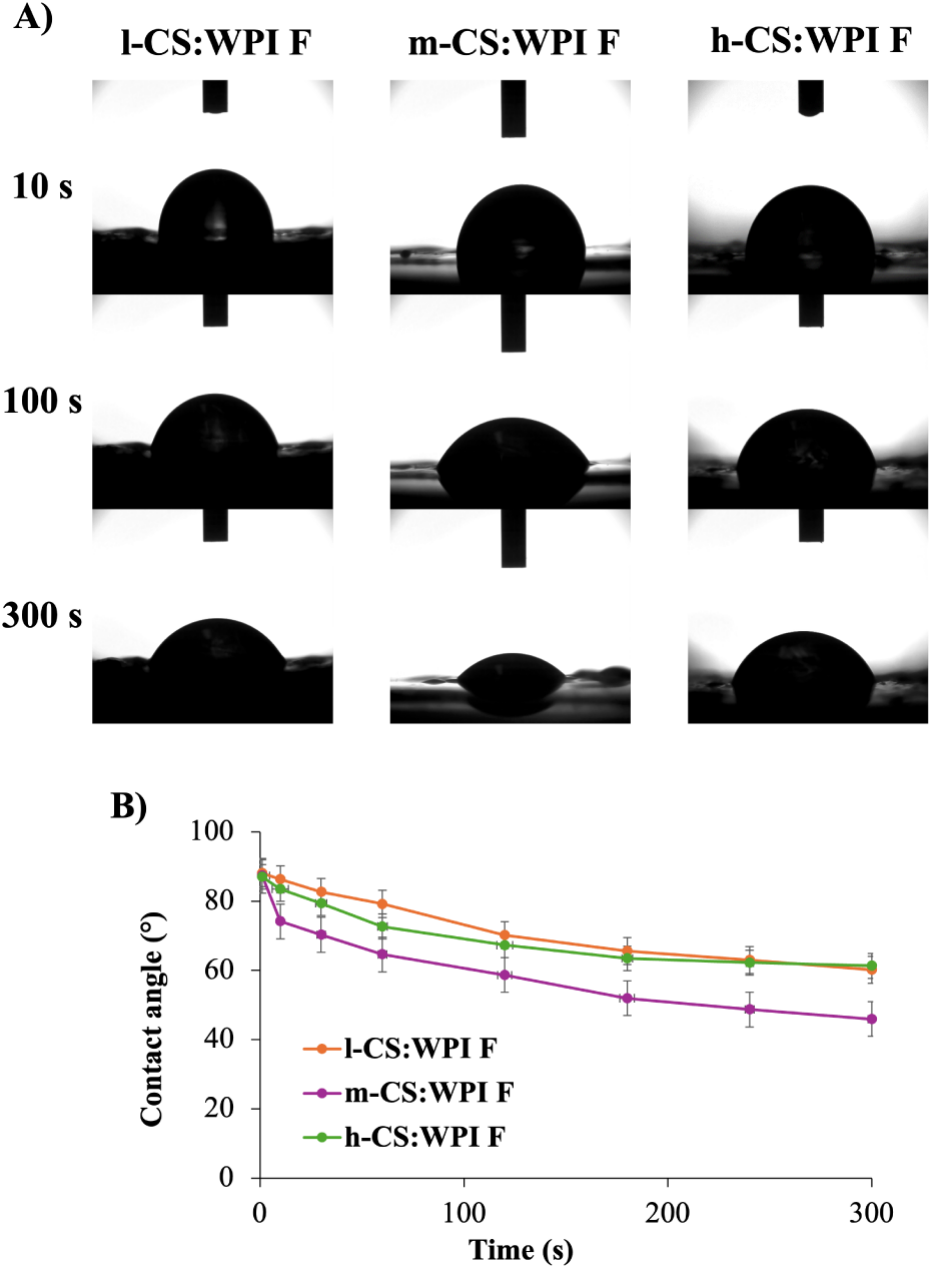
A) Representative images acquired during contact angle measurements for l-CS:WPI F, m-CS:WPI F, and h-CS:WPI F films at 10, 100, and 300 s. B) Comparison of contact angle values of the three CS:WPI films up to 300 s (mean values ± SD; *n* = 3).

Degradation studies were carried out both in PBS and PBS supplemented with H_2_O_2_ in order to mimic wound bed conditions. Given the biodegradable nature of the films, degradation rather than rapid dissolution represents the most relevant process under physiological conditions. The degradation medium was supplemented with H_2_O_2_ to mimic the oxidative microenvironment of the wound bed, where reactive oxygen species are physiologically produced by inflammatory cells and are known to influence biomaterial stability and degradation.^45,46^

As shown in Figure 8A, the residual mass % of the three films at 1, 3, and 7 days of incubation indicates that all samples underwent an initial sharp degradation, reaching residual mass % values of about 50% in both PBS and PBS + H_2_O. After 3 and 7 days no further or only minimal degradation was observed for all samples in both degradation media. Among the samples, m-CS-based films exhibited the highest resistance to degradation, consistent with their stronger protein–polysaccharide interactions. Conversely, h-CS:WPI film was the most susceptible to degradation, probably due to its more fragile structure, as previously evidenced by characterization studies.

**Figure 8:**
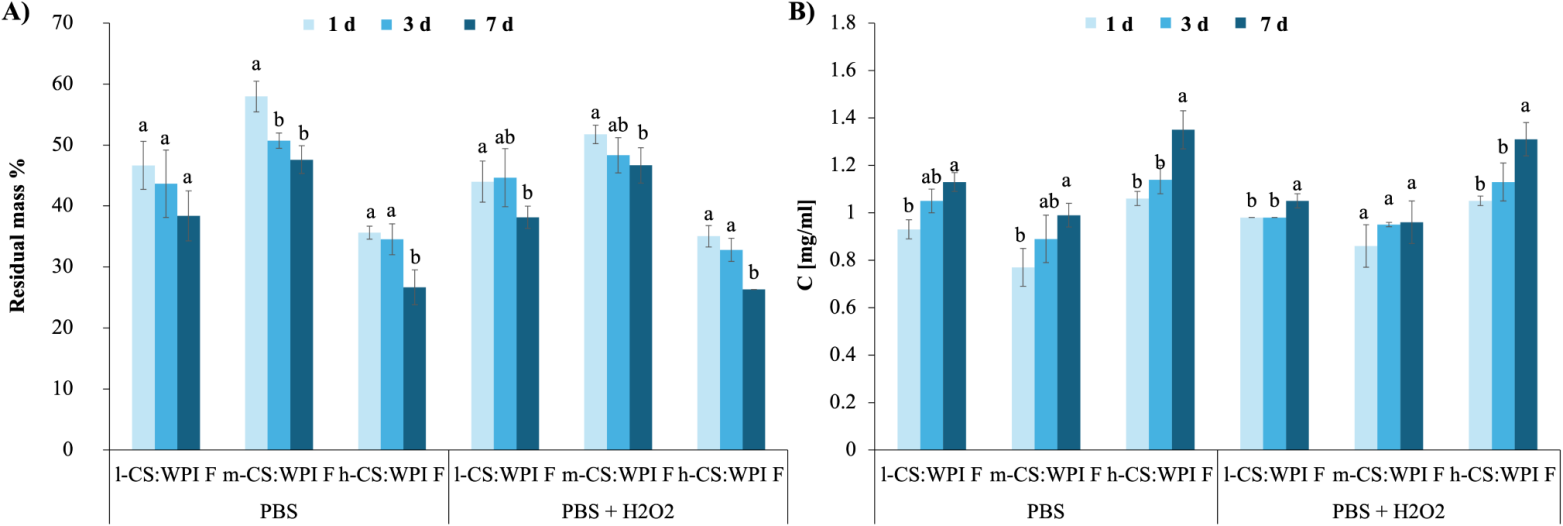
A) Residual mass (%) of CS:WPI films after degradation in PBS and PBS + H_2_O_2_ at 32 °C for 1, 3, and 7 days. B) WPI concentration released in the degradation media at the different time points, determined by UV–Vis spectrophotometric analysis at 280 nm. Data are expressed as mean ± SD (*n* = 3). Statistical analysis was performed by one-way ANOVA followed by Scheffé post hoc test. Different letters indicate statistically significant differences among time points within the same formulation and degradation medium (*p* < 0.05), whereas shared letters indicate no significant differences.

To confirm the results obtained by the gravimetric method, spectrophotometric analysis at 280 nm was performed on the degradation media after 1, 3, and 7 days of contact with the films to quantify the concentration of dissolved proteins. Figure 8B shows the WPI concentrations measured in the media after exposure to l-CS:WPI, m-CS:WPI, and h-CS:WPI films. A progressive increase in WPI concentration over time was observed for all sample, in good agreement with the gravimetric analysis.

In vitro studies were subsequently carried out to evaluate the cytocompatibility and antimicrobial activity of films.

Indirect cytotoxicity tests performed on NHDF confirmed the biocompatibility of all formulations, with cell viability values comparable to those of the control medium (Figure 9A). Interestingly, the film containing m-CS significantly enhanced fibroblast proliferation, suggesting a stimulatory effect on cell growth (Figure 9B). This feature is particularly advantageous for wound dressings, as it supports the re-epithelialization process and accelerates tissue regeneration. The results of the in vitro wound healing assay performed on NHDF are reported in Figure 9C and are expressed as the percentage reduction of wound area over time (min). All films were able to promote cell migration to a similar or greater extent compared to the control (CM), confirming their ability to support tissue regeneration. No significant differences among groups were observed during the early time points, while m-CS:WPI progressively showed the lowest residual gap area, indicating the most pronounced promotion of NHDF migration. At later time points, m-CS:WPI was significantly different from all other groups, supporting the positive effect of the optimized CS/WPI interaction on fibroblast-mediated wound closure. This trend is consistent with the proliferation results, indicating that the optimal interaction between m-CS and WPI provides a favorable microenvironment for both cell migration and proliferation. Conversely, l-CS- and h-CS-based films showed a less marked effect; the h-CS-based film performed better than the CM, while the l-CS-based film showed comparable results to the CM. These findings confirm that CS MW plays a crucial role in modulating the cellular response.

**Figure 9:**
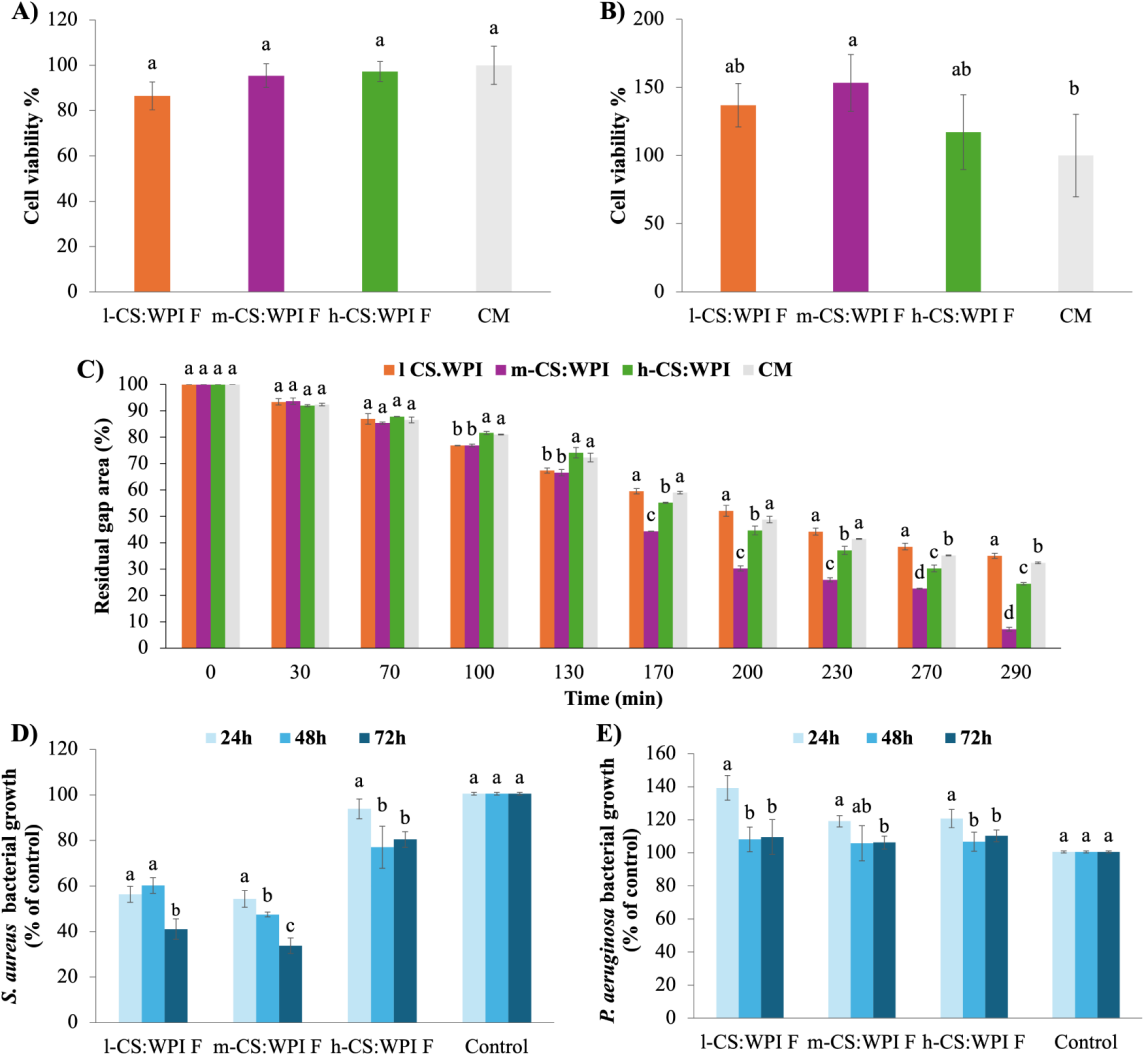
A) Cytocompatibility results expressed as Cell viability (%) of NHDF after exposure to conditioned medium (mean ± SD; *n* = 6). B) Proliferation results expressed as Cell viability (%) of NHDF after exposure to conditioned medium (mean ± SD; *n* = 6). C) Wound healing assay on NHDF cells expressed as Residual gap area (%) over Time (min) (mean ± SD; *n* = 3). D) Antibacterial activity against S.aureus after exposure to conditioned medium for 24, 48, and 72 h. (mean ± SD; *n* = 3). E) Antibacterial activity against P.aeruginosa after exposure to conditioned medium for 24, 48, and 72 h (mean ± SD; *n* = 3). Statistical analysis was performed by one-way ANOVA followed by Scheffé post hoc test. Different letters indicate statistically significant differences among samples within the same experimental condition or time point (*p* < 0.05), whereas shared letters indicate no significant differences.

Moreover, the antibacterial activity of the CS:WPI films against *S. aureus* and *P. aeruginosa* is reported in Figures 9D and 9E, respectively. Overall, all CS:WPI F exhibited an evident inhibitory effect against *S. aureus*, confirming the intrinsic antimicrobial potential of the developed formulations. This outcome was expected, considering the intrinsic antimicrobial properties of both CS and WPI.^47–49^ Regarding the influence of CS MW, a correlation with antibacterial efficiency was observed, consistent with the trends previously reported for other functional and biological properties. Films containing m-CS demonstrated the highest antibacterial activity, followed by those based on l-CS and h-CS. This result is partially in contrast with what reported in literature, where l-CS is often reported to exert the strongest antibacterial effect due to its higher solubility and the shorter polymer chains, enhancing stronger electrostatic interactions between protonated amino groups and negatively charged bacterial cell surfaces.^50–52^ In the present study, however, the films containing m-CS showed the most pronounced antibacterial effect. This finding suggests that the synergistic interaction between CS and WPI reaches its maximum effectiveness when CS is employed at medium-MW, further confirming the pivotal role of MW in modulating the overall behavior of the films, as already observed for other characterizations. Moreover, it is noteworthy that both CS and WPI retained their intrinsic antimicrobial properties within the composite matrix, indicating that their interaction does not hinder, but rather preserves the overall antimicrobial activity of the system.

In contrast, when tested against *P. aeruginosa*, the CS:WPI films prepared with all CS MW did not exhibit a significant antibacterial effect (Figure 9E). The percentages of viable cells remained close to those of the control at all time points, indicating a limited inhibition of bacterial growth. This behavior can be attributed to the intrinsic resistance mechanisms of *P. aeruginosa*, a Gram-negative bacterium characterized by an outer membrane rich in lipopolysaccharides that hinders the penetration of large or positively charged molecules such as CS. Furthermore, the lower surface charge density of *P. aeruginosa* reduces electrostatic interactions with the protonated amino groups of CS, further limiting its activity.^53,54^

Finally, the in vivo wound healing properties of films were assessed in a murine model. It should be noted that the in vivo assay was carried out in accordance with blinding procedures: an independent researcher (unaware of the nature of the samples) was responsible of animal treatments and a separate operator, also blinded to the sample identities, was in charge of histological evaluation. Histological analysis of skin biopsies collected on day 18 revealed clear differences between treated and control wounds, as reported in Figure 10A. H&E staining showed that, in the saline-treated group (negative control), wound regeneration was incomplete, with persistent inflammatory infiltrate, a thin epidermis, and a disorganized dermal matrix. Conversely, all films-treated groups exhibited improved tissue regeneration compared to the negative control. Interestingly, the group treated with m-CS:WPI film showed complete epidermal reconstruction, with thickness and morphology comparable to healthy skin. The dermis appeared well-organized, with an extracellular matrix rich in mature collagen, as highlighted by PSR staining. Moreover, a reduced presence of inflammatory cells was observed compared to the other groups. The group treated with l-CS:WPI film demonstrated good regeneration; however, the epidermis was thinner and collagen less organized than in the m-CS:WPI group. Finally, h-CS:WPI film also demonstrated satisfactory regeneration, but with some irregularities in epidermal thickness and a slightly less homogeneous dermal matrix. In addition, residual wound area was quantified from photographs acquired at each time point and reported as residual wound area (%) in Figure 10C. Significant differences were observed among treatments, particularly at later time points. The m-CS:WPI-treated wounds showed the lowest residual wound area, supporting the enhanced wound healing performance of this formulation. Furthermore, after 18 days of treatment, the wound area was completely free of any film residues, indicating full in vivo biodegradation of the materials. This finding is particularly relevant, as an essential requirement for an ideal wound dressing is that its degradation rate should match the tissue regeneration process.^55^

**Figure 10:**
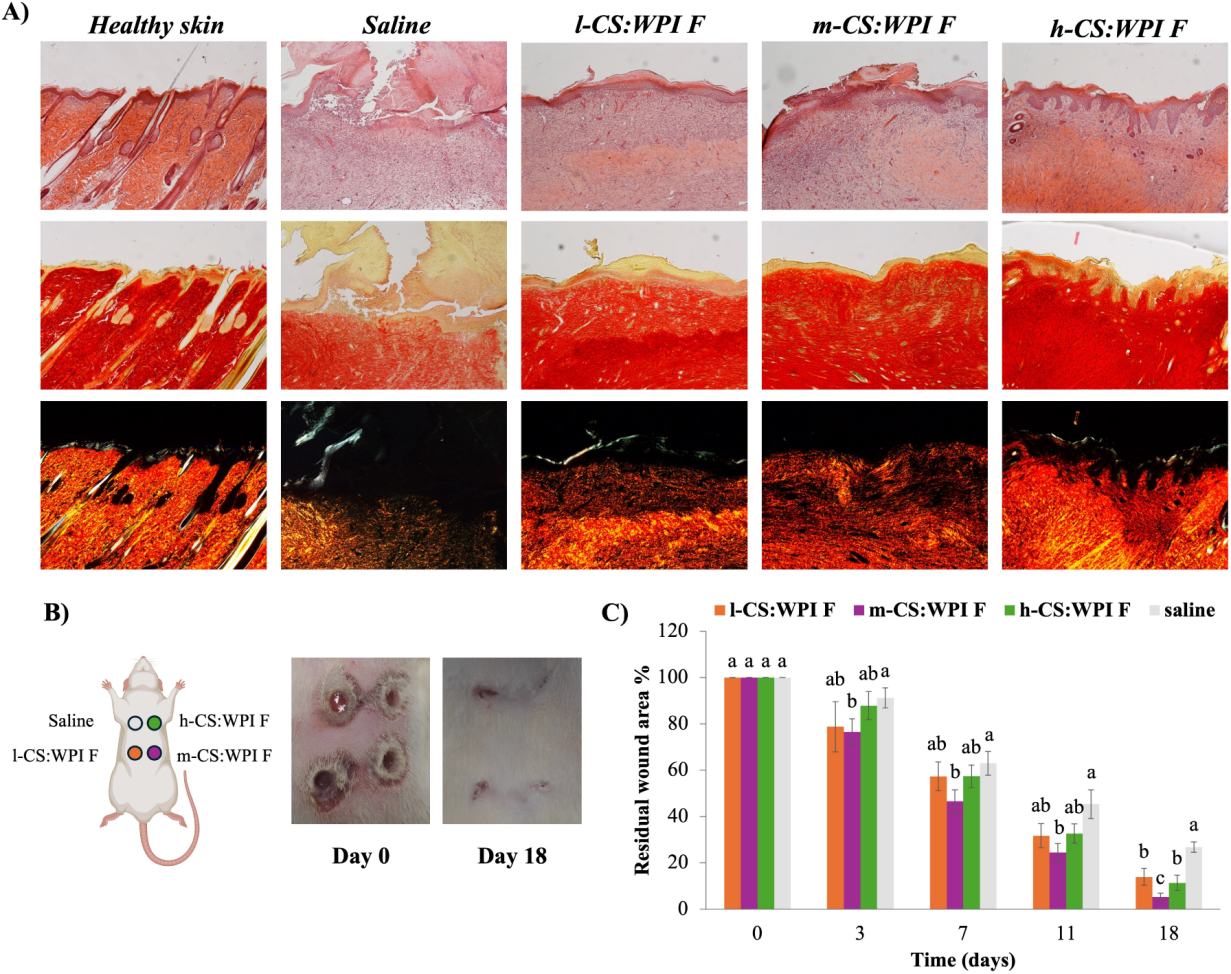
A) Histological evaluation of wound healing 18 days after treatment. Representative images of skin sections stained with H&E (top row), picrosirius red (PSR) under bright-field microscopy (middle row), and PSR under polarized light (bottom row) for: healthy skin (nontreated control), saline-treated wound (negative control), and wounds treated with l-CS:WPI, m-CS:WPI, and h-CS:WPI films. B) Schematic representation of the disposition of the different samples and the control (saline) on rat back (left) and photographs of rat back taken on day 0 and day 18 of treatment. C) Residual wound area (%) values (mean ± SD; *n* = 3) calculated by measuring wound area with ImageJ on the images taken at the specific time points (Day 0, Day 4, Day 7, Day 11 and Day 18). Statistical analysis was performed by one-way ANOVA followed by Scheffé post hoc test: different letters indicate statistically significant differences among treatments at the same time point; whereas shared letters indicate no significant differences.

These results suggest that the presence of CS with different MW significantly influences the quality of skin regeneration after wound treatment. Among the tested formulation, the m-CS-containing film proved to be the most effective in promoting the formation of newly generated tissue structurally similar to healthy skin, supporting mature collagen deposition and reducing the inflammatory response. These findings indicate that CS/WPI-based films represent a promising strategy for the development of advanced biomaterials for skin wound healing.

To contextualize the performance of the optimized m-CS:WPI film within the current state of the art, representative natural-polymer-based wound dressing films were selected from the literature and compared in Table 3 in terms of mechanical properties, swelling/water uptake, degradation, antibacterial and in vivo wound healing outcomes. Only film-like systems were considered, whereas hydrogels, electrospun fibers, sponges, and three-dimensional scaffolds were excluded. Due to the methodological heterogeneity among published wound dressing studies, including differences in materials, testing conditions, bacterial strains, and in vivo models, the comparison was not intended as a direct quantitative ranking. Instead, literature data were used to define representative benchmark ranges for key parameters relevant to advanced wound management, allowing the performance of the proposed films to be contextualized within the current state of the art. In particular, the results highlight that the proposed system exhibits a well-balanced combination of mechanical, hydration, antibacterial, and in vivo regenerative properties.

**Table 3:** Comparison of the Optimized M-CS:WPI Film with Representative Natural-Polymer-Based Wound Dressing Films

Film composition	Mechanical properties	Swelling/water uptake	Degradation	Antibacterial activity	In vivo wound healing	Reference
m-CS:WPI	TS: ∼2.7 MPa; EB: ∼100%	>150%, 24 h	Residual mass: ∼50% after 7 d	Strong inhibition vs *S. aureus*; limited effect vs *P. aeruginosa*	Residual wound area: ∼5% at day 18; complete epidermal reconstruction	
Chitosan/gelatin + tannic acid and/or bacterial nanocellulose	TS: 81.83–102.45 MPa; EAB: 2.89–7.52%	Approx. 3000–4000%	n.r.	n.r.	Approx. 80–90% wound contraction at day 15	^56^
Chitosan/CMC/tannic acid/beeswax	TS: 0.275 ± 0.003 MPa	283.0 ± 2.0%, 2 h	n.r.	antibacterial efficiency: 80.8% vs *S. aureus*	90.0 ± 3.3% burn wound healing; 88.85 ± 1.7% infected wound healing at day 7	^11^
Chitosan + Hypericum perforatum oil	TS: 44.6–14.8 MPa; EAB:	176–115%	n.r.	Inhibition zone: 1.24–1.97 cm vs *S. aureus*; 2.00–2.93 cm vs *E. coli*	n.r.	^57^
Sodium alginate + Moringa oleifera extract/essential oil	TS: 0.248 MPa; EAB: 31.41%	4500–1800%, 20 min	n.r.	n.r.	n.r.	^58^

## Conclusion

CS/WPI-based films were successfully developed as advanced biobased wound dressings through a formulation-driven approach. The systematic investigation of different CS MWs and CS:WPI ratios highlighted the superior performance of m-CS:WPI F (1:3 ratio), which showed the best balance in terms of mechanical properties, hydration capacity (>150%), stability (residual mass ∼50% after 7 days). In vitro assays confirmed that all CS:WPI F are biocompatible, able to promote NHDF proliferation and migration, and endowed with antimicrobial activity especially against *S. aureus*. Among the tested formulations, m-CS:WPI film exhibited the most pronounced biological activity, significantly supporting cell proliferation and enhancing wound closure. In vivo studies further confirmed its ability to accelerate wound regeneration (residual wound area ∼5%), with complete epidermal reconstruction, organized collagen deposition, reduced inflammatory infiltrate, and full biodegradation after 18 days.

Importantly, this work establishes a correlation between polymer–protein interactions, quantified through rheological synergism, and the resulting functional and biological performance of the material. This finding supports the use of rheological synergism in the rational design of polymer–protein-based biomaterials.

Overall, these findings demonstrate that CS:WPI films, and especially the m-CS-based formulation, represent promising candidates for the development of advanced wound dressings, combining structural integrity, antimicrobial activity, and pro-regenerative properties for wound healing applications.
